# Intracellular Nitric Oxide and cAMP Are Involved in Cellulolytic Enzyme Production in *Neurospora crassa*

**DOI:** 10.3390/ijms24054503

**Published:** 2023-02-24

**Authors:** Nan-Nan Yu, Wirinthip Ketya, Gyungsoon Park

**Affiliations:** 1 Plasma Bioscience Research Center, Department of Plasma-Bio Display, Kwangwoon University, Seoul 01897, Republic of Korea; 2 Department of Electrical and Biological Physics, Kwangwoon University, Seoul 01897, Republic of Korea

**Keywords:** *Neurospora crassa*, cellulase, enzyme production, nitric oxide, cyclic AMP, MAPK, filamentous fungi, enzyme activity, signaling pathways, reactive oxygen species

## Abstract

Although molecular regulation of cellulolytic enzyme production in filamentous fungi has been actively explored, the underlying signaling processes in fungal cells are still not clearly understood. In this study, the molecular signaling mechanism regulating cellulase production in *Neurospora crassa* was investigated. We found that the transcription and extracellular cellulolytic activity of four cellulolytic enzymes (*cbh1*, *gh6-2*, *gh5-1*, and *gh3-4*) increased in Avicel (microcrystalline cellulose) medium. Intracellular nitric oxide (NO) and reactive oxygen species (ROS) detected by fluorescent dyes were observed in larger areas of fungal hyphae grown in Avicel medium compared to those grown in glucose medium. The transcription of the four cellulolytic enzyme genes in fungal hyphae grown in Avicel medium was significantly decreased and increased after NO was intracellularly removed and extracellularly added, respectively. Furthermore, we found that the cyclic AMP (cAMP) level in fungal cells was significantly decreased after intracellular NO removal, and the addition of cAMP could enhance cellulolytic enzyme activity. Taken together, our data suggest that the increase in intracellular NO in response to cellulose in media may have promoted the transcription of cellulolytic enzymes and participated in the elevation of intracellular cAMP, eventually leading to improved extracellular cellulolytic enzyme activity.

## 1. Introduction

Cellulose is the most abundant renewable resource in nature and has broad application prospects [1]. The enzyme cellulase can decompose cellulose into soluble reducing sugars and is an essential component of cellulose industrial applications in diverse fields ranging from the pulp and paper industry to winemaking and renewable energy power generation [2]. According to reports of global enzyme market analyses, cellulases are the third most important industrial enzymes, with an exponentially increasing demand [3].

Filamentous fungi are widely used for producing cellulolytic enzymes [4]. Because filamentous fungi can secrete extracellular proteins with high efficiency, the extracellular activity of fungal cellulolytic enzymes tends to be higher than that of bacterial cellulolytic enzymes [5]. Fungi, as eukaryotic organisms, have advanced mechanisms for post-translational processing of proteins, such as glycosylation, protease cleavage, and disulfide bond formation, which are critical for conferring specific functions on cellulolytic enzymes [6]. Cellulolytic enzymes have been reported to be produced by many filamentous fungi, such as *Penicillium oxalicum*, *Aspergillus niger*, *Trichoderma reesei* and *Neurospora crassa* [7], among which *T. reesei* is the most actively studied and widely used fungal species in industrial cellulase production. Additionally, *N. crassa* has been actively explored for cellulolytic enzyme production [8,9]. Fungi secrete three main types of cellulolytic enzymes, namely endoglucanase (endo-1, 4-β-D-glucanase, EG, EC 3.2.1.4), cellobiohydrolase or exoglucanase (exo-1, 4-β-D-glucanase, CBH, EC 3.2.1.91), and β-glucosidase (1, 4-β-D-glucosidase, BG, EC 3.2.1.21), which generally act synergistically to degrade cellulose [5]. The content of these three cellulolytic enzymes has been shown to reach 69% of the total secreted protein under the induction of Avicel (microcrystalline cellulose) medium in *N. crassa* [10]. In addition, *N. crassa* can also secrete accessory proteins, such as GH61 family proteins (lytic polysaccharide monooxygenase) and CDH-1 (cellobiose dehydrogenase), which synergistically promote the hydrolysis of cellulose with cellulolytic enzymes [10]. Production of cellulolytic enzymes is regulated mostly at the transcription level in *N. crassa*, and a protein kinase, STK-12, is known to act as a transcriptional brake in the expression of cellulase-encoding genes [11]. Transcription factors such as cellulose degradation regulator 1/2 (Clr-1/2), activator of cellulase expression 1/2/3 (Ace 1/2/3), xylanase regulator 1 (Xyr1), carbon catabolite repressor (Cre1) and beta-glucosidase regulator (BglR) have been also identified as transcriptional regulators in the expression of cellulolytic enzymes in *N. crassa* [12].

Fungal cellulolytic enzyme production is regulated by complex molecular mechanisms. Studies have shown that *N. crassa* can sense the cellobiose concentration in its environment and transport cellobiose into cells through cellodextrin transporters (CDT) on the cell membrane, and finally activate the cellulase transcription factor to promote the expression of cellulolytic enzymes [8,13,14]. A recent study has reported a novel regulatory strategy for cellulase production, in which G-protein signaling activates the cellulase transcription factor by regulating cAMP levels [15]. In *T. reesei*, multiple signaling pathways, such as cAMP/PKA, calcium, and MAPK, are involved in the regulation of cellulase formation [16,17,18,19,20,21,22,23,24,25]. In addition, changes in components of signaling pathways, such as small GTPase, MAPK, Ca^2+^, and polyphosphoinositol, in the presence of cellulose were identified in *N. crassa* through sequencing and bioinformatics analysis [26]. Multiple signaling pathways may regulate the production of fungal cellulolytic enzymes, and there may be a tandem relationship between these pathways.

Molecular regulatory networks for fungal cellulase production are not completely understood and still have many gaps that need to be elucidated. In this study, we investigated the molecular basis of the transcription and extracellular activity of cellulolytic enzymes, particularly focusing on clarifying the internal signaling pathways, in *N. crassa*. As a natural degrader of cellulose, *N. crassa* has been considered as a model organism in many studies to explore the regulatory mechanisms of cellulolytic genes [27]. We used Avicel^®^ PH-101 (microcrystalline cellulose with a size of approximately 50 μm) as an inducer of cellulase production in this study. Avicel is often used to induce mRNA transcription and protein secretion of cellulolytic enzymes in *N. crassa* [8,10]. To explore the production mechanism of fungal cellulolytic enzymes, cellulose is usually added as the sole carbon source to induce the expression of cellulolytic enzymes. In the presence of glucose, a fungus preferentially uptakes glucose from the environment, and the production of cellulolytic enzymes is inhibited via carbon catabolite repression [28]. Media for cellulase induction (Avicel) or non-induction (glucose) may be quite different, even in their physicochemical properties, such as conductivity, acidity, alkalinity, and redox capacity, because of significantly different molecular weights and solubilities between cellulose and glucose. The changes in these physicochemical properties can act as signals for activating signaling pathways within fungal hyphae. In this study, we also analyzed the differences in the physicochemical properties of media and their relationship with the production of fungal cellulolytic enzymes.

## 2. Results

### 2.1. Avicel Induces Cellulase Production in N. crassa

**Figure 1:**
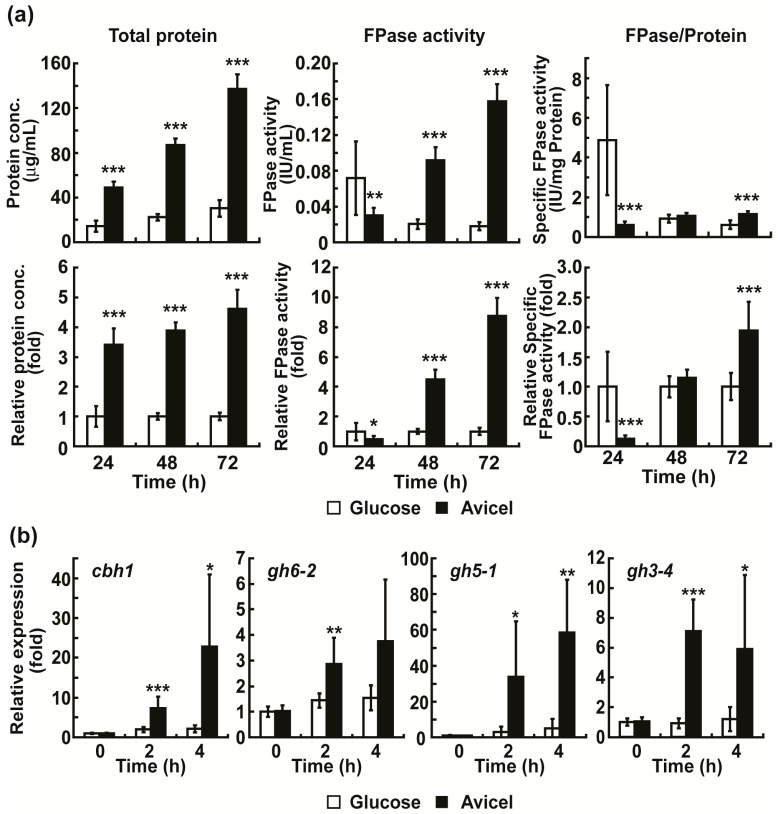
Analysis of activity and mRNA expression of cellulolytic enzymes. (**a**) FPase activity, total protein concentration, and specific enzyme activity were measured in media containing Avicel or glucose after 24, 48, and 72 h, and the relative fold changes between Avicel and glucose media were calculated. (**b**) mRNA levels of four cellulolytic enzymes (*cbh1*, *gh6-2*, *gh5-1*, and *gh3-4*) in fungal hyphae grown in media containing Avicel or glucose after 2 and 4 h. Each value is the mean of six or nine replicate measurements (three replicates per experiment and two or three independent experiments): * *p* < 0.05, ** *p* < 0.01, *** *p* < 0.001 as determined by Student’s *t*-test.

Cellulolytic enzymes in *N. crassa* are produced when celluloses are provided as the only carbon source [8]. We also confirmed this in our experimental conditions. The filter paper-degrading activity (FPase activity, total activity of cellulolytic enzymes) and total protein concentration were measured when Avicel or glucose (control; no induction of cellulases) was provided as the carbon source in media. The FPase activity increased significantly in the medium containing Avicel after 48 h (346.98 %) and 72 h (774.72 %), while no significant change in activity was observed in the medium containing glucose (Figure 1a). The total protein concentration in Avicel medium was significantly increased after 24 h (240.14 %), 48 h (288.26 %), and 72 h (361.47 %), compared to no significant change in glucose medium (Figure 1a). Specific enzyme activity (total enzyme activity divided by total protein) was increased in Avicel medium only after 72 h (94.36 %) (Figure 1a).

High levels of extracellular enzymes may result from the increased intracellular expression of enzyme proteins. To test this hypothesis, we measured the mRNA levels of four cellulolytic enzymes, including two cellobiohydrolases (*cbh1* and *gh6-2*), an endoglucanase (*gh5-1*), and a β-glucosidase (*gh3-4*), known to be abundantly secreted upon Avicel induction [10]. A significant increase in the mRNA levels of the four cellulolytic enzyme genes was observed in the hyphae grown in Avicel media after 2 h (at least 2.5 times higher compared to that at 0 h) and 4 h (at least 3 times higher compared to that at 0 h), compared to the uninduced hyphae (grown in glucose containing media) (Figure 1b).

**Figure 2:**
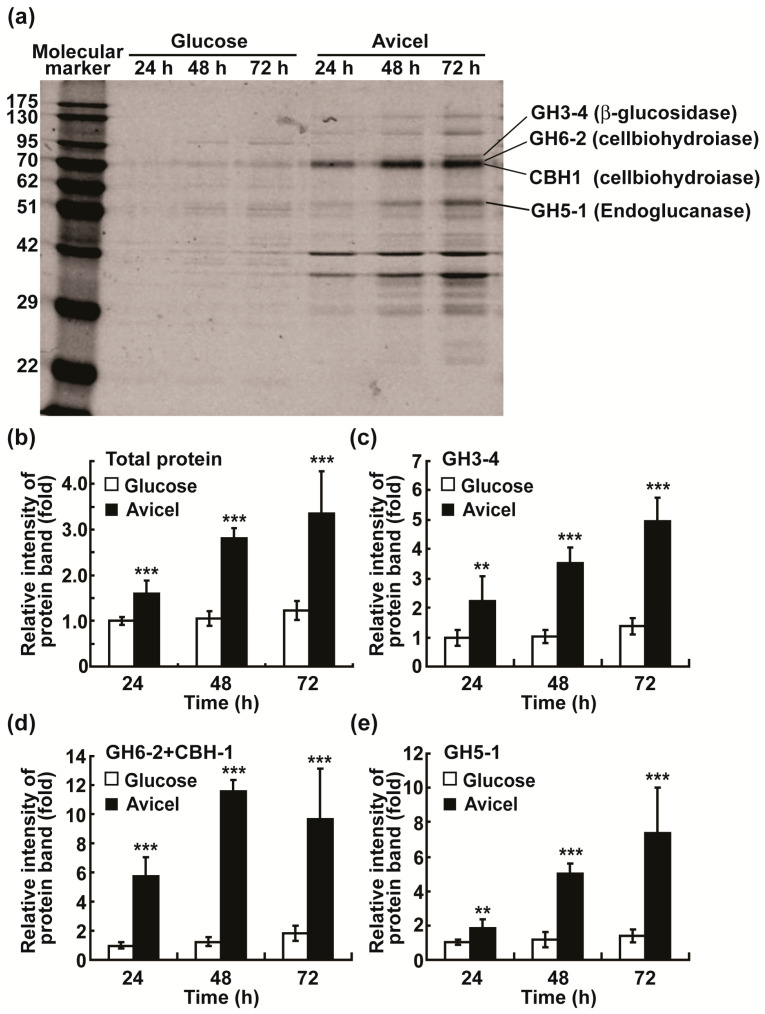
Analysis of secreted cellulolytic enzymes in media after 24, 48, and 72 h. (**a**) SDS-PAGE of secreted proteins in media. The gel was stained with Coomassie Blue R-250. Bands corresponding to four cellulolytic enzymes are indicated. (**b**–**e**) The band intensity of cellulolytic enzymes quantified using the ImageJ software (National Institute of Health, Bethesda, MD, USA). The relative intensity of the total of four enzyme bands (**b**), GH3-4 band (**c**), GH6-2 + CBH1 band (**d**) or GH5-1 band (**e**) was estimated as a ratio in band intensity compared to those in the glucose group at 24 h. Each value is the mean of six replicate measurements (three replicates per experiment and two independent experiments): ** *p* < 0.01, *** *p* < 0.001 as determined by Student’s *t*-test.

Furthermore, we estimated the protein levels of the four cellulolytic enzymes in media by analyzing their protein profiles using SDS (sodium dodecyl sulfate)–PAGE (polyacrylamide gel electrophoresis). Protein band intensity was generally higher in Avicel medium than in glucose medium (Figure 2a), and the four protein bands corresponding to each cellulolytic enzyme were identified according to their molecular weights as previously described [29]. Total intensity of the four enzyme bands estimated using the ImageJ software was significantly higher in the samples collected from Avicel medium than in those collected from glucose medium after 24 h, 48 h and 72 h (Figure 2b). Intensity of GH3-4 or GH5-1 was significantly higher in Avicel than in glucose media at all incubation times (Figure 2c,e). Because bands of GH6-2 and CBH1 proteins were not separated on the gel, the intensity of a band including both proteins was estimated. It was significantly greater in Avicel than in glucose media at all incubation times (Figure 2d).

### 2.2. Physicochemical Properties Were Different between Glucose and Avicel Media

**Figure 3:**
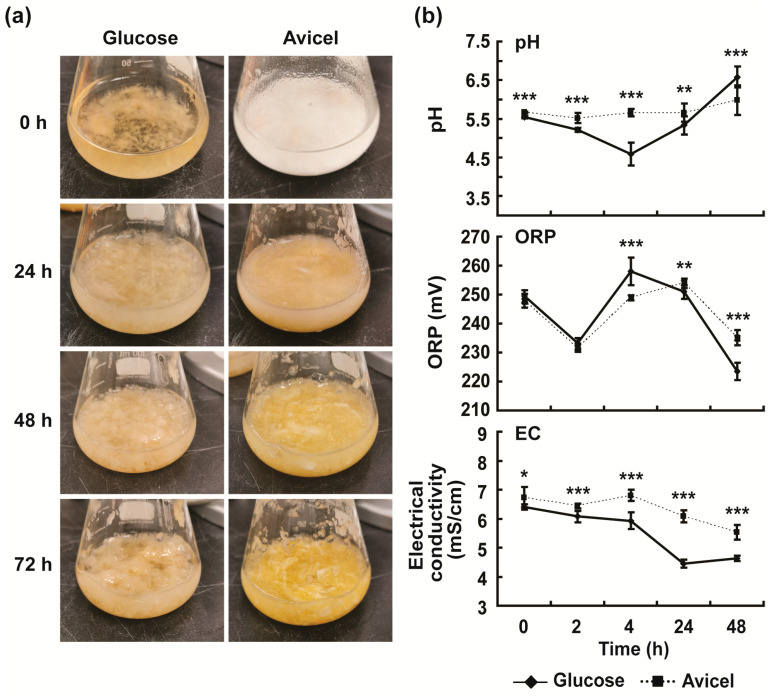
Physical and chemical properties of media during incubation. (**a**) Photographs of *Neurospora crassa* cultured in glucose or Avicel medium. (**b**) pH, ORP and EC of media. The media were harvested after 0, 2, 4, 24, 48, and 72 h. Each value represents the mean of 9 or 12 replicate measurements (three replicates per experiment and three or four independent experiments): * *p* < 0.05, ** *p* < 0.01, *** *p* < 0.001 as determined by Student’s *t*-test.

To find whether changes in the extracellular media environment affected the expression and secretion of cellulolytic enzymes in *N. crassa*, the physical and chemical properties of both glucose and Avicel media were analyzed during incubation. The white precipitate in Avicel medium (Avicel is a water-insoluble cellulose polymer) gradually disappeared as the incubation time increased (Figure 3a), indicating that celluloses were degraded into soluble sugars by cellulolytic enzymes before they could be used by cells. The pH of Avicel medium did not change dramatically during incubation, but remained significantly higher than that of glucose medium during 0–24 h incubation (5.52–5.66 vs. 4.59–5.55) (Figure 3b). At 48 h incubation time, the pH of Avicel medium was significantly lower than that of glucose medium (5.99 vs. 6.58) (Figure 3b). The oxidation–reduction potential (ORP) value of Avicel medium was lower than that of glucose medium during 0–4 h incubation (231.56–248.89 vs. 233.22–258.11 mV; significantly lower at 4 h incubation), and significantly higher than that of glucose medium at 48 h incubation (235.11 vs. 223.44 mV) (Figure 3b). Generally, the changes in pH values in both media were contrary to the changes in ORP values during incubation (Figure 3b). This accords with the previous findings that pH is an important factor causing the change in the ORP value: the higher the pH, the lower the ORP [30]. The electrical conductivity (EC) gradually decreased as the incubation time increased in both glucose and Avicel media, although no dramatic decrease was observed during 0–4 h incubation (Figure 3b). At all incubation times, including 0 h, the EC values were significantly higher in Avicel medium than in glucose medium (Figure 3b). Solvent concentration is positively related to the EC of the solution [31].

### 2.3. Intracellular NO and ROS Levels Significantly Increased in N. crassa Cultured in Avicel Medium

**Figure 4:**
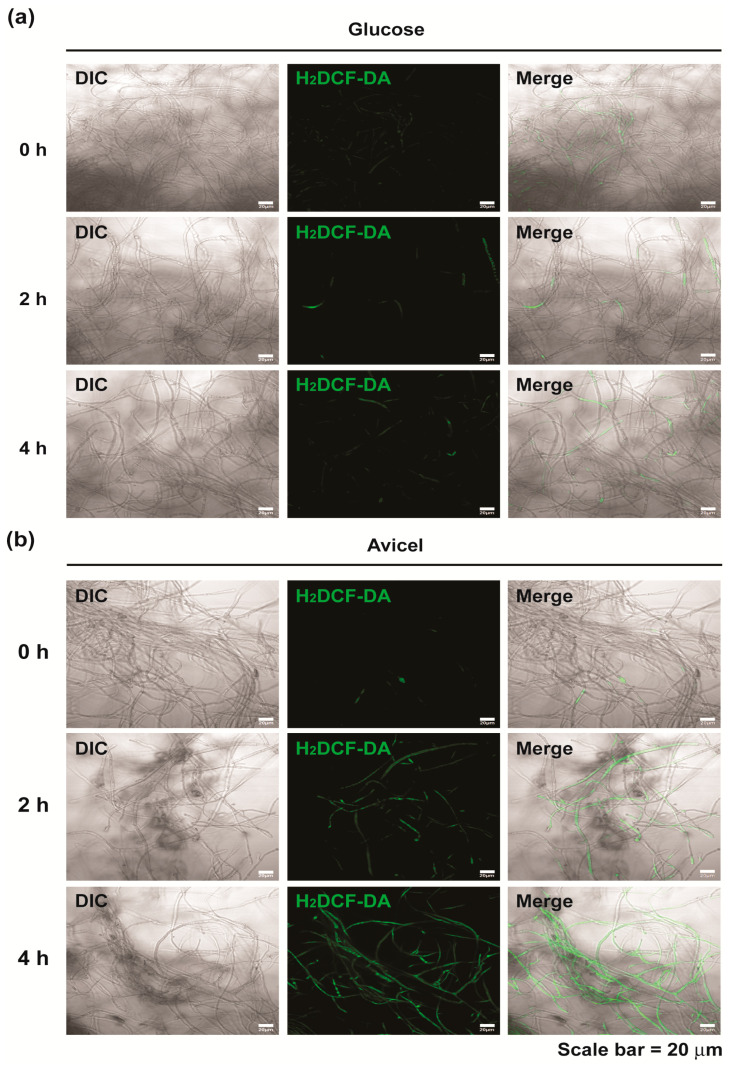

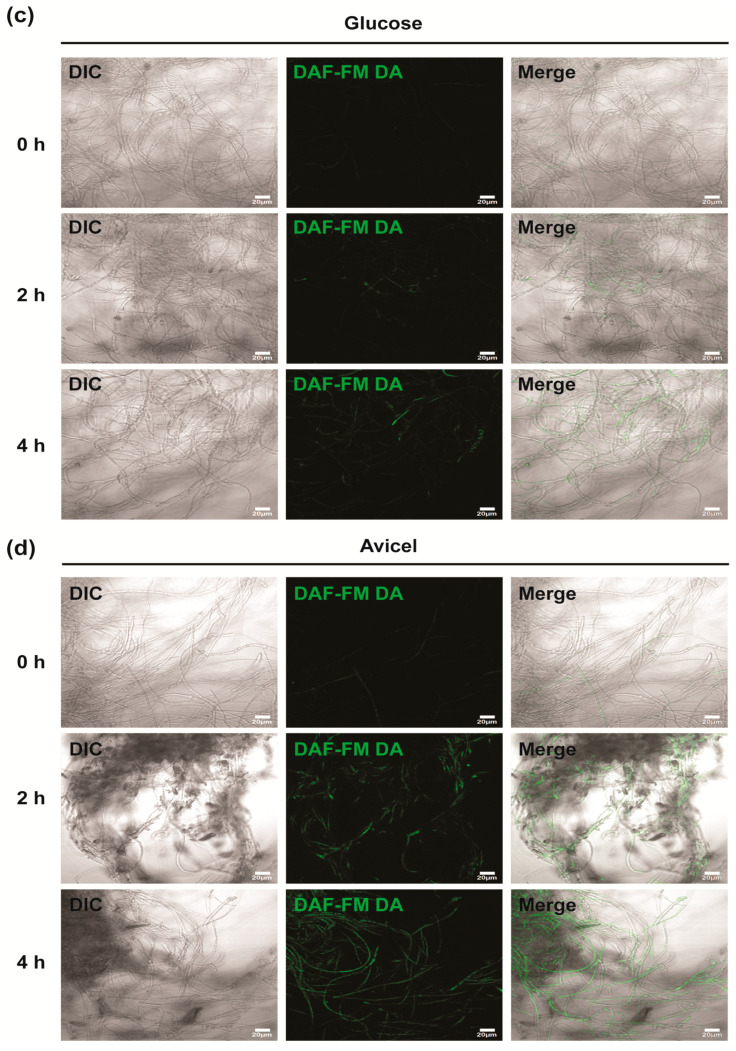
Analysis of intracellular ROS and NO in *Neurospora crassa* hyphae grown for 0, 2 and 4 h. Intracellular ROS was detected using H_2_DCF-DA (green fluorescence) in fungal hyphae grown in glucose (**a**) or Avicel (**b**) medium. Intracellular NO was detected using DAF-FM DA (green fluorescence) in fungal hyphae grown in glucose (**c**) or Avicel (**d**) medium. DIC: differential interference contrast, H_2_DCF-DA or DAF-FM DA: fluorescence, Merge: combined image of DIC and fluorescence.

An ORP value represents the redox capacity of a solution, and the redox capacity of the culture environment may be related to the generation of intracellular reactive oxygen and nitrogen species (RONS) [32]. To test this, we detected the intracellular NO and ROS using fluorescent dyes in fungal hyphae grown in both media. The intensity of ROS and NO fluorescence was found to be stronger in fungal hyphae grown in Avicel medium (induced) than in those grown in glucose (non-induced) medium after 2 h and 4 h incubation (Figure 4 and Supplementary Figure S1). However, no significant change in ROS and NO production in fungal cells was observed between glucose and Avicel media after 24 and 48 h (Supplementary Figure S2). Fluorescence was hardly detected in most samples (Supplementary Figure S2). These results indicate that intracellular ROS and NO production significantly increased in medium containing Avicel only during the early incubation period (0–4 h).

### 2.4. Intracellular NO Is Involved in the Regulation of Cellulolytic Enzyme Production in Avicel Medium

**Figure 5:**
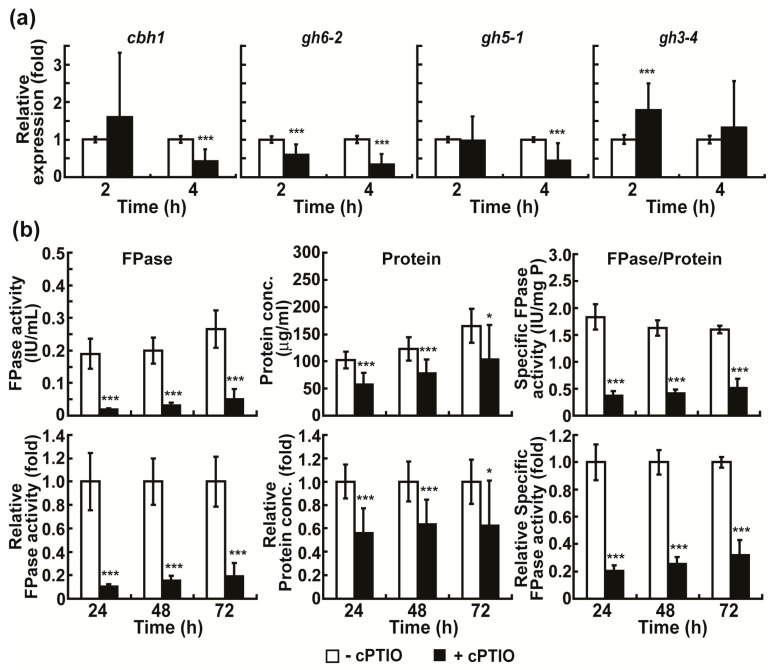
Effect of cPTIO treatment on cellulolytic enzyme production. (**a**) mRNA levels of cellulolytic enzymes (*cbh1*, *gh6-2*, *gh5-1*, and *gh3-4*) in fungal hyphae grown in Avicel medium with or without cPTIO for 2 and 4 h. (**b**) FPase activity (total activity of cellulolytic enzymes), total protein concentration, and specific enzyme activity measured in media after fungus was grown in Avicel medium with or without cPTIO for 24, 48, and 72 h. −cPTIO group: only Avicel medium, +cPTIO group: 10 mM cPTIO in Avicel medium. Each value is the mean of 9 or 12 replicate measurements (three replicates per experiment and three or four independent experiments): * *p* < 0.05, *** *p* < 0.001 as determined by Student’s *t*-test.

The elevated production of intracellular ROS and NO in fungal hyphae grown in Avicel medium during early incubation suggested their potential role in the production (mostly gene expression) of cellulolytic enzymes. To validate this, we first analyzed the role of intracellular NO in cellulolytic enzyme production in Avicel medium. This is because experimental tools, such as scavengers and donors, are available only for NO, and peroxynitrite anion (ONOO^−^), a highly reactive species formed by a rapid reaction between nitric oxide (NO) and superoxide anion, is highly sensitive to H_2_DCF-DA [33]. After the application of the NO scavenger, 2-4-carboxyphenyl-4,4,5,5-tetramethylimidazoline-1-oxyl-3-oxide (cPTIO), the levels of *cbh1*, *gh6-2*, *and gh5-1* mRNA significantly decreased in fungal hyphae cultured in Avicel medium, mostly after 4 h (Figure 5a). After 24–72 h, the total cellulolytic activity, total protein concentration, and specific cellulolytic activity were significantly reduced in Avicel medium containing cPTIO (Figure 5b). The highest decrease was observed after 24 h (Figure 5b).

**Figure 6:**
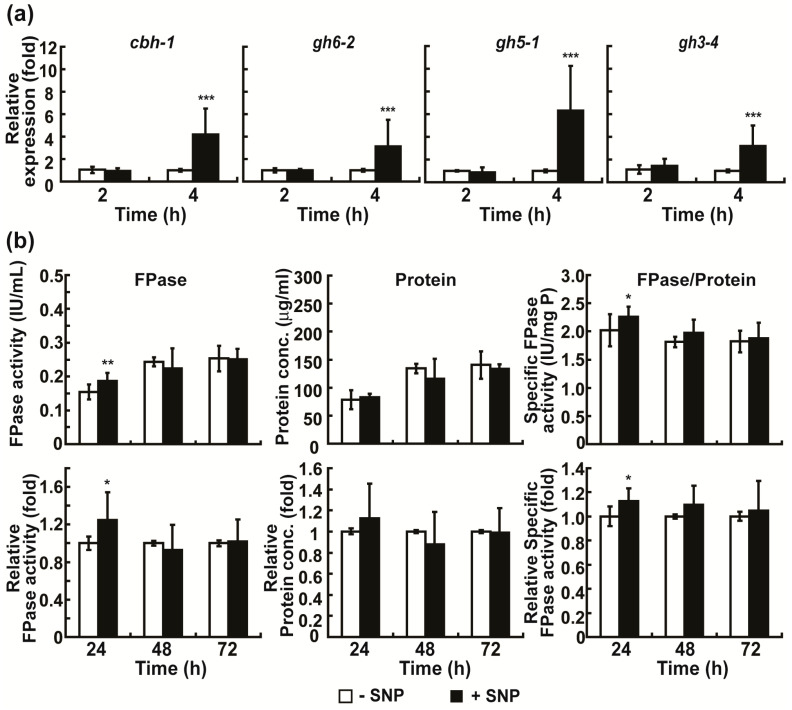
Effect of sodium nitroprusside (SNP) treatment on cellulolytic enzyme production. (**a**) mRNA levels of cellulolytic enzymes (*cbh1*, *gh6-2*, *gh5-1*, and *gh3-4*) in fungal hyphae grown in Avicel medium with or without SNP for 2 and 4 h. (**b**) FPase activity (total activity of cellulolytic enzymes), total protein concentration, and specific enzyme activity measured in media after fungus was grown in Avicel medium with or without SNP for 24, 48, and 72 h. −SNP group: only Avicel medium, +SNP group: 0.01 mM SNP in Avicel medium. Each value is the mean of nine replicate measurements (three replicates per experiment and three independent experiments): * *p* < 0.05, ** *p* < 0.01, *** *p* < 0.001 as determined by Student’s *t*-test.

When the NO donor, sodium nitroprusside (SNP), was added to Avicel medium (0.01 mM), the mRNA levels of *cbh1*, *gh6-2*, *gh5-1*, and *gh3-4* were significantly increased in fungal hyphae grown in this medium after 4 h (Figure 6a). Generally, no significant difference in total cellulolytic activity, protein concentration, and specific enzyme activity in media was observed between the control (no SNP) and SNP treatment after 24, 48, and 72 h; however, an approximately 24.59% increase in total enzyme activity was observed in the SNP-treated group after 24 h (Figure 6b).

Notably, the transcription of *gh6-2*, *gh5-1*, and *gh3-4* was significantly elevated in glucose medium after the addition of SNP, mainly after 2 h (Supplementary Figure S3a). The total cellulolytic activity (filter paper-degrading activity) of glucose medium was also slightly increased, mainly after 24 h in the SNP-treated sample (Supplementary Figure S3b). This result is quite intriguing and warrants further investigation because cellulolytic enzymes are generally not induced in glucose medium.

We examined whether cPTIO and SNP treatments affected fungal growth. No significant change in the dry weight of the fungal mycelia grown in Avicel medium was observed after cPTIO or SNP treatment at all incubation times (Supplementary Figure S4a,b). In glucose medium, the dry weight of the harvested fungal mycelia was not significantly different between the control and SNP treatment at all incubation times (Supplementary Figure S4c). Thus, we concluded that cPTIO and SNP treatments may not affect the fungal growth.

### 2.5. Intracellular NO May Be Closely Associated with cAMP Signaling in the Regulation of Cellulolytic Enzyme Production

**Figure 7:**
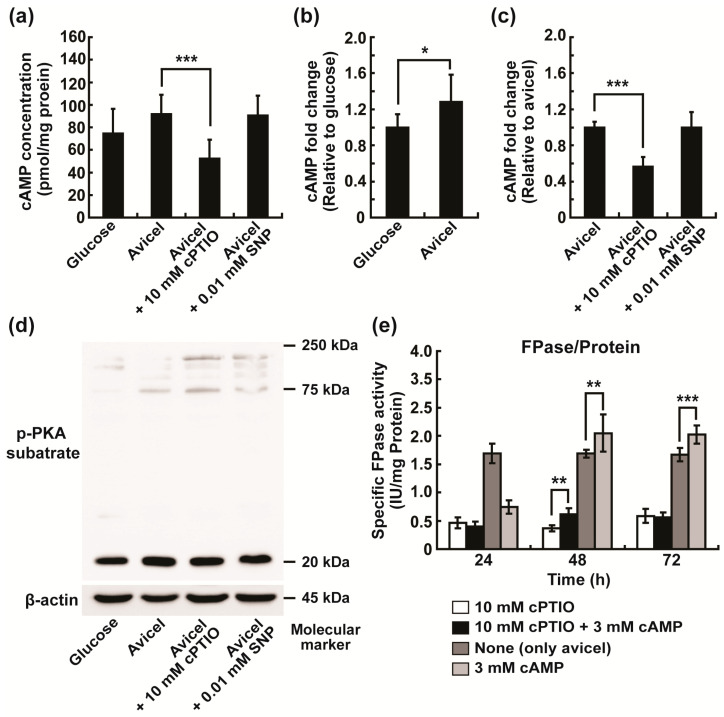
Analysis of the relationship between intracellular NO and cAMP signaling. (**a**–**c**) Measurement of intracellular cAMP concentration; cAMP concentration in fungal hyphae grown in glucose or Avicel medium for 4 h and effects of cPTIO and SNP on cAMP concentration (**a**), fold change in cAMP concentration compared to that in glucose medium (**b**), fold change in cAMP concentration compared to that in Avicel medium (**c**). (**d**) Levels of phospho-PKA substrate detected by Western blot to reflect PKA activity. (**e**) Specific activity of cellulolytic enzymes (activity of cellulolytic enzymes per mg of secreted protein) measured in Avicel medium after 24, 48, and 72 h. Each value represents the mean of six or nine replicate measurements (three replicates per experiment and two or three independent experiments): * *p* < 0.05, ** *p* < 0.01, *** *p* < 0.001 as determined by Student’s *t*-test. cPTIO: 2-4-carboxyphenyl-4,4,5,5-tetramethylimidazoline-1-oxyl-3-oxide; SNP: sodium nitroprusside.

In filamentous fungi, cAMP is known to activate cAMP-dependent protein kinase A (PKA) and regulate cellulase gene transcription [15,18]. To clarify how intracellular NO can regulate the production of cellulolytic enzymes, we examined the possibility of the involvement of cAMP signaling. We first measured the cAMP concentration and PKA activity in fungal hyphae grown in Avicel medium for 4 h because intracellular NO was increased after 2–4 h as shown in Figure 4d. Intracellular cAMP concentration was slightly higher in fungal hyphae grown in Avicel medium than in glucose medium, significantly decreased with the addition of cPTIO (removal of intracellular NO), and not significantly changed with the addition of SNP (Figure 7a).

When relative ratios were calculated and compared using the same data, the cAMP level in fungal hyphae grown in Avicel medium was increased by approximately 28% compared to that in glucose medium (Figure 7b). The cPTIO treatment caused an approximately 43.33% decrease in the intracellular level of cAMP in Avicel medium (Figure 7c). We assessed PKA activity in fungal hyphae grown in glucose or Avicel medium for 4 h by Western blot analysis of phosphorylated peptides or proteins (as a result of PKA action). No significant difference in band number and intensity (indication of PKA activity) was observed between glucose and Avicel media and between control and cPTIO and SNP treatments in Avicel medium (Figure 7d).

To further analyze the roles of intracellular NO and cAMP in extracellular cellulase production, we added cPTIO and cAMP to fungal culture and measured the cellulolytic activity in media after 24–72 h. The results showed that cPTIO addition reduced the activity of cellulolytic enzymes in Avicel medium, and the addition of cAMP in the presence of cPTIO could slightly rescue cellulolytic activity after 48 h, but not after 24 and 72 h (Figure 7e). When cAMP was added to the fungal culture in Avicel medium, the activity of cellulolytic enzymes was significantly elevated after 48 and 72 h (Figure 7e), indicating that the intracellularly produced NO is likely to accelerate the intracellular production of cellulases, possibly by inducing cAMP production, which in turn may lead to the elevation of extracellular cellulolytic activity.

Calcium signaling is known to promote cellulase mRNA transcription via *crz1* (calcineurin-responsive zinc finger transcription factor 1) in *T. reesei* [17,34]. We stained Ca^2+^ using the fluorescent dye, Fluo-3 AM, after 2 and 4 h in *N. crassa* hyphae and found no significant difference in fluorescence between glucose and Avicel cultures (Supplementary Figure S5). This suggests that calcium signaling may not be related to Avicel-induced increase in NO.

In addition, MAP kinases are known to be involved in cellulase formation in *T. reesei* [35]. We assessed the activation of these MAP kinases in *N. crassa* (Fus3 homolog; MAK-2, Slt2 homolog; MAK-1, Hog1 homolog; OS-2) during the elevation of intracellular NO in hyphae by examining the phosphorylation of MAP kinases. Our preliminary results showed no significant difference in phosphorylated OS-2 levels among glucose and Avicel media and with the addition of cPTIO and SNP (Supplementary Figure S6). Many bands were detected by anti-phospho-p44/42 antibody, and we estimated two bands corresponding to MAK-1 and MAK-2 by analyzing the molecular weights of these two MAP kinases. The phosphorylation levels of MAK-1 and MAK-2 seemed to increase slightly after the addition of cPTIO (Supplementary Figure S6), indicating a possibility of the negative role of MAK-1 and MAK-2 in the production of cellulases, as shown in *T. reesei* [35,36].

## 3. Discussion

Regulatory networks involved in the production of fungal cellulases have been continuously studied and reported to include changes in multiple signals. However, the mechanisms by which these intracellular signaling pathways are activated and the tandem relationship among these signals are not completely understood. As frequently demonstrated in previous studies, our study verified that external signals, such as cellulose, initiated signal transduction, leading to the transcription of cellulolytic enzyme genes and further extracellular cellulolytic activity [13,24].

Notably, this initial cellulose signal seems to induce the generation of secondary intracellular signals, such as ROS and NO, in *N. crassa* cells, as shown by our data. We observed that the intracellular ROS and NO levels increased in response to the presence of cellulose in media. These signals seem to be involved in regulating the transcription of cellulolytic enzyme genes rather than the extracellular secretion of cellulolytic enzymes because intracellular ROS and NO levels were elevated only during early incubation (2–4 h), not during later incubation (24–48 h; secretion stage). We further analyzed the effect of NO in this study, just because NO scavenger and donor are available and can be easily used in experiments. Our results showed that the transcription of cellulolytic enzyme genes significantly changed with the addition of cPTIO (NO scavenger) or SNP (NO donor); the removal of intracellular NO significantly decreased the transcription of cellulolytic enzyme genes, leading to the decrease in extracellular cellulolytic enzyme activity, and the addition of NO extracellularly increased the expression of cellulolytic enzyme genes. This elucidated the positive regulatory role of intracellular NO in *N. crassa* cellulase production. Intracellular ROS and NO production during fungal cellulase production has been rarely observed. Hydrogen peroxide (H_2_O_2_) is generated in fungal co-cultures and is responsible for the increase in laccase and manganese peroxidase activities [37]. A recent study showed that the level of intracellular ROS detected using H_2_DCFDA in *T. reesei* increased after Sr^2+^ was supplemented in media, and the intracellular ROS was detrimental to cellulase production [38]. H_2_DCFDA used for detecting intracellular ROS in our study was more sensitive to peroxinitrite (ONOO^−^) and hydroxyl radical (OH·) than to other species, and peroxinitrite is formed by a rapid reaction between NO and superoxide anion (O_2_·^−^) [33]. This indicates that H_2_DCFDA-mediated detection indirectly reflects the presence of intracellular NO. Taken together, our results suggest a novel, hitherto unknown mechanism by which extracellular cellulose (Avicel) induces NO production within fungal cells and intracellular NO plays a positive regulatory role in the expression of fungal cellulase genes.

Intracellular NO generated in response to Avicel seemed to be related to the elevation of cAMP, a well-known secondary messenger in fungal cells, in our study. The cAMP level significantly decreased together with extracellular specific cellulolytic enzyme activity when intracellular NO was removed in Avicel medium. Addition of cAMP into Avicel medium slightly increased the cellulolytic enzyme activity, and the addition of cAMP together with cPTIO (NO scavenger) slightly restored the reduced cellulase activity. Many studies reported the involvement of intracellular cAMP in fungal cellulase production [15,18,21].

Regarding signaling pathways regulated by cAMP or ROS/NO for cellulase production, the cAMP/PKA pathway, calcium signaling, and MAPK signaling can be considered because the involvement of these signaling pathways has been frequently reported in *T. reesei*. The cAMP/PKA pathway has been known to be essential for regulating the expression of cellulase-encoding genes through activation or inactivation of transcription factors CRE1, ACE1, CLR1/2, and HAP2/3/5 complex in *T. reesei* [18,21]. In *N. crassa*, cAMP signaling serves as a downstream target of G-protein GNA-3, regulating cellulose degradation [15]. In our study, we observed no significant change in substrate protein phosphorylation by cAMP-dependent PKA after NO removal or addition. This indicates that cAMP generation in response to the elevation of intracellular NO level may not lead to the activation of PKA but be involved in other cellular pathways to control cellulase production.

Calcium and MAPK signaling pathways have been actively explored in the production of *T. reesei* cellulases [16,17,19,20,22,23,24,25]. Recently, cAMP was found to activate calcium signaling for regulating cellulase production in *T. reesei* [22]. MAPK signaling also regulates cellulase production, and Hog1-like MAPK plays a positive role in the transcription of cellulase-encoding genes [16]. Fus3- and Slt2-like MAP kinases negatively regulate cellulase formation indirectly by repressing growth and maintaining cell wall integrity [35]. In this study, several preliminary analyses were performed to determine if Ca^2+^ and MAPK signaling are associated with intracellular NO or cAMP levels that induce cellulase production. Our results showed that the Ca^2+^ level in Avicel medium was not significantly different from that in glucose medium, indicating that Ca^2+^ signaling may not be associated with cellulase production. In addition, no significant change was detected in the phosphorylation of Hog1-like MAPK (OS-2) in Avicel medium, and intracellular NO removal or addition did not seem to significantly affect the level of phosphorylation of this MAPK. Phosphorylation of Fus3- and SLt2-like MAPKs (MAK-1 and MAK-2) seemed to slightly increase after NO removal in Avicel medium, suggesting the suppression of MAK-1 and MAK-2 activities under the elevated intracellular NO condition in response to cellulose in media, which may possibly indicate the negative role of MAK-1 and MAK-2 in cellulase production in *N. crassa*, as shown in *T. reesei*. Further in-depth investigation of this issue may be needed.

Interestingly, physical signals can play a major role in the induction of cellulase formation. Physical environmental signals, such as light and pH, have been suggested as induction signals for cellulase production besides cellulose in *T. reesei* [24]. The ORP, pH, and EC in the culture environment can affect various physiological activities of microorganisms [39,40]. Light-sensing photoreceptors activate cAMP and calcium signaling pathways, regulating cellulase expression [22,41]. The receptor PAC1 activates cellulase production through the pal signaling pathway in a neutral pH environment [42]. A decrease in intracellular pH stimulates Ca^2+^ input through the Ca^2+^ channel and increases cellulase transcription through the Ca^2+^ signaling pathway [17]. In our experiments, light was continuously applied to fungus, and pH was generally higher in Avicel than in glucose medium during incubation. Notably, the EC values were higher in Avicel than in glucose medium during all incubation times. Different electrical properties of media may affect the fungal cell membrane and membrane transport characteristics, such as opening of channel or transporter proteins on membrane. Studies in *T. reesei* have shown that cellobiose released from cellulose degradation may be a direct signal because cellobiose, not cellulose, can be transported into cells [24]. Cellobiose transport through a membrane transporter can be a limiting factor for the initiation of cellulase expression. Several sugar transporters involved in the regulation of cellulase production have been identified in *T. reesei* [24]. The transporter-mediated control of sugar transport is likely critical for inducing cellulase production. Change in membrane electrical properties caused by the electrical environment in media may possibly affect the efficiency of a transporter.

## 4. Materials and Methods

### 4.1. Fungal Strain and Culture Conditions

*Neurospora crassa* (strain name: ORS-SL6a, mating type: mat a, FGSC number: 4200) was used in this study and obtained from the Fungal Genetics Stock Center (FGSC, Manhattan, KS, USA). The fungus was maintained on Vogel’s Minimal (VM) agar. Fungal culture in glucose and Avicel media was performed as follows: a piece of *N. crassa* mycelia was inoculated onto VM agar media in a flask and cultured at 30 °C in darkness for 2 d and then at 25 °C in light for 12 d. Sterile deionized (DI) water (approximately 50 mL) was added into the flask and then shaken vigorously. Fungal suspension was filtered through two layers of Miracloth (EMD Millipore, Burlington, MA, USA) and then centrifuged at 3134× *g* for 5 min to pellet down spores. The spore pellet was resuspended in fresh sterile DI water. Spores (3 × 10^7^) were inoculated into 30 mL VM liquid with 2% (*w*/*v*) glucose and incubated at 25 °C under constant light with shaking (200 rpm) for 24 h. Fungal mycelia were then collected by filtration through two layers of Miracloth (EMD Millipore) and washed with DI water. The washed mycelia were resuspended in 30 mL of fresh VM containing 2% (*w*/*v*) glucose or 2% (*w*/*v*) Avicel (Avicel PH-101, Sigma-Aldrich, St. Louis, MI, USA) and incubated at 25 °C under constant light with shaking (200 rpm) for 24, 48, and 72 h.

### 4.2. Measurement of EC, ORP, and pH in the Media

The culture medium was collected at different incubation time points and centrifuged at 2390× *g* for 10 min, and the supernatant was recovered for measuring EC, ORP, and pH. The ORP was measured using an ExStik^TM^ Model RE300 waterproof ORP meter (Extech, Nashua, NH, USA), and the pH was measured using a portable pH meter (Oakton Instruments, Vernonb Hills, IL, USA). The EC was measured using a PCTSTestr™ 50 Waterproof Pocket pH/Cond/TDS/Salinity Tester (Oakton Instruments, Vernon Hills, IL, USA). All measurements were performed according to the manufacturer’s instructions.

### 4.3. Assay for Enzymatic Activities and Protein Concentration

To measure protein concentration and cellulase activity in the medium, the culture medium was harvested at 24, 48, and 72 h after Avicel induction and then centrifuged at 2390× *g* for 10 min. The supernatant was collected and stored at 4 °C. Within 48 h, total protein concentration in the supernatant was determined using the Bradford protein assay kit (Bio-Rad, Hercules, CA, USA) according to the manufacturer’s protocol. Bradford solution (1×, 200 μL) was added into 10 μL culture supernatant, and then the mixture was incubated at 25 °C for 5 min in the dark. The absorbance of solution was measured at 595 nm using a microplate reader (Biotek, Winooski, VT, USA).

Total activity of cellulolytic enzymes was determined by the rate of degradation of filter paper (FPase), as described previously [43]. Briefly, a reaction mixture containing Whatman filter paper no. 1. (substrate; Sigma-Aldrich, St. Louis, MI, USA), 30 μL 0.1 M acetate buffer (pH 5.6) and 30 μL culture supernatant was incubated at 50 °C for 30 min. Levels of liberated reducing sugars (product of the enzymatic reaction) were measured by adding 120 μL 3,5-dinitrosalicylic acid (DNS; Sigma-Aldrich, St. Louis, MI, USA) into the reaction mixture and then boiling for 10 min. After 720 μL of deionized water was added to the reaction mixture, the solution absorbance was measured at 540 nm using a microplate reader (Biotek). One unit of enzymatic activity was defined as the amount of enzyme capable of producing 1 μM of reducing sugars from the appropriate substrates per minute. Specific enzyme activity was estimated by dividing the total enzyme activity by the total protein amount.

### 4.4. Protein Gel Electrophoresis and Western Blotting

Culture media were collected at 24, 48, and 72 h after Avicel induction, and hyphae were removed by centrifugation at 2390× *g* for 10 min. The supernatant was mixed with sample buffer (5×), and the mixture was boiled for 5 min. A 20 μL solution (30 µg total protein) was applied to a sodium dodecyl sulfate -polyacrylamide (12%) gel, and the gel was electrophoresed. Gels were stained overnight with Coomassie Blue R-250 (Bio-Rad). The protein bands corresponding to relevant cellulolytic enzymes were determined according to their molecular weights [29]. Photography and analysis were performed using a ChemiDoc^TM^ MP imaging system (Bio-Rad) and ImageJ software version 1.52a (National Institute of Health).

For Western blot analysis, mycelia were collected after Avicel induction for 4 h and ground with a mortar and pestle under liquid nitrogen. Protein concentration was determined using the Bradford protein assay kit (Bio-Rad). The same amount of protein (30 µg/20 μL) was subjected to SDS-polyacrylamide gel (12 %) electrophoresis and electro-transferred to a nitrocellulose membrane (Millipore, Bedford, MA, USA). The membrane was blocked with 5% milk, washed three times with 1× PBS, and then incubated with the primary antibody (1:1000 dilution) at 4 °C overnight. Primary antibodies used in the study were as follows: anti-Hog1 (D-3) (sc-165978; Santa Cruz Biotechnology, Santa Cruz, CA, USA), anti-p44/42 MAPK (#9102; Cell Signaling Technology, Danvers, MA, USA), anti-phospho-p38 MAPK (#9211; Cell Signaling), anti-phospho-p44/42 MAPK (#9101; Cell Signaling), anti-PKA substrate antibody (#9621; Cell Signaling), and anti-β-actin (#4967; Cell Signaling). After washing with 1× PBS, the membrane was incubated with anti-rabbit IgG, HRP-conjugated secondary antibody (1:2500 dilution; Thermo Fisher, Rockford, IL, USA) for 1 h at 25 °C. Finally, Clarity Western ECL Substrate (Bio-Rad) was added onto the washed membrane, and chemiluminescence was detected using a ChemiDoc^TM^ MP imaging system (Bio-Rad). Analysis was performed using ImageJ software version 1.52a (National Institute of Health).

### 4.5. Quantitative Real-Time PCR Analysis

Quantitative real-time PCR was used to analyze the mRNA expression levels of the four cellulolytic enzymes. Fungal mycelia were collected by filtration through Miracloth (EMD Millipore) at 0, 2, and 4 h after Avicel induction and immediately frozen in liquid nitrogen and stored at −80 °C until analysis. RNAiso Plus (TaKaRa Bio, Shiga, Japan) was used to extract total RNA, and ReverTra Ace qPCR RT Master Mix with gDNA Remover (Toyobo, Osaka, Japan) was used to synthesize cDNA, according to the manufacturer’s instructions. Real-time PCR was performed using the iQ SYBR Green Supermix (Bio-Rad) and CFX 96^TM^ real time Instrument (Bio-Rad), following the manufacturer’s instructions. The thermal cycling conditions were 95 °C for 3 min, 40 cycles at 95 °C for 10 s, and 60 °C for 30 s. Primer sequences are listed in Table 1. *β-actin* was used as a reference gene. Cycle threshold (Ct) values were determined, and the mRNA levels for each enzyme were normalized to the reference gene (*β-actin*). The relative mRNA level of enzyme gene in each incubation time was calculated based on the difference in Ct values compared to that of the glucose-treated sample at 0 h as follows: 2^−∆∆Ct^, where ∆∆Ct = (Ct_target_ − Ct_reference_) Avicel at all incubation times or glucose at 2 and 4 h—(Ct_target_ − Ct_reference_) glucose at 0 h [44].

### 4.6. Fluorescence Staining

To detect intracellular ROS and NO, fungal mycelia were harvested at 0, 2, 4, 24, 48 h after incubation in glucose or Avicel medium. The harvested mycelia were stained with 20 μM 2′,7′-dichlorodihydrofluorescein diacetate (H_2_DCF-DA, Themo Fisher, Waltham, MA, USA) and 20 μM 4-amino-5-methylamino-2′,7′-difluorofluorescein diacetate (DAF-FM DA, Themo Fisher) for intracellular ROS and NO, respectively, at 25 °C in the dark for 1 h.

Intracellular Ca^2+^ was detected using the fluorescent dye Fluo3-AM (Invitrogen, Carlsbad, CA, USA). Fungal mycelia were harvested at 0, 2, 4, 24, 48 h after incubation in glucose or Avicel medium and then stained with 5 μM of Fluo3-AM (Invitrogen) at 25 °C in the dark for 1 h.

After incubation, all stained samples were washed with 1× PBS and then imaged using a FV-100 MPE spectral confocal laser scanning microscope (Olympus Corporation, Tokyo, Japan) under wavelengths corresponding to each fluorescent dye: 488/525 nm (excitation/emission) for H_2_DCFDA, 495/515 nm (ex./em.) for DAFFMDA, 506/526 nm (ex./em.) for Fluo3-AM.

### 4.7. cPTIO (NO Scavenger), SNP (NO Donor), and cAMP Treatment

For cPTIO (2-(4-carboxyphenyl)-4,5-dihydro-4,4,5,5-tetramethyl-1 H-imidazolyl-1-oxy-3-oxide), SNP (sodium nitroprusside), or cAMP (adenosine 3′,5′-cyclic monophosphate sodium salt monohydrate) treatment, 1 × 10^6^ spores of *N. crassa* were inoculated into 1 mL VM liquid with 2% (*w*/*v*) glucose in a 24-well plate. After being incubated at 25 °C for 24 h, the fungal mycelia were washed twice with DI water, and 1 mL of VM liquid with 2% (*w*/*v*) Avicel supplemented with 10 mM cPTIO (Calbiochem, San Diego, CA, USA), 0.01 mM SNP (Sigma-Aldrich), or 3 mM cAMP (Sigma-Aldrich) was added. The culture was incubated under constant light at 25 °C with shaking (200 rpm).

### 4.8. cAMP Concentration Measurement

cAMP concentrations were determined using a cAMP Competitive ELISA Kit (Invitrogen) according to the manufacturer’s protocol. Fungal mycelia were harvested at 4 h and immediately triturated with liquid nitrogen, and PBS was added into ground fungal powder. Subsequently, the mixture was centrifuged at 600× *g* for 10 min, and the supernatant was collected and stored at −80 °C until analysis. Neutralizing reagent was added into the supernatant, and then cyclic AMP antibody, cyclic AMP-AP conjugate solution, substrate solution, and stop solution were added in sequence according to the manufacturer’s instructions. Finally, the absorbance of the resulting solution was measured at 450 nm using a microplate reader (Biotek).

### 4.9. Statistical Analysis

All data are presented as the mean ± standard deviation (SD) from at least six replicates. Paired Student’s *t*-tests and two-way analysis of variance were performed, followed by Tukey’s post hoc test. A *p*-value < 0.05 was considered to reflect a statistically significant difference. SPSS Statistics Software, version 25 (IBM, Chicago, IL, USA) was used for statistical analysis.

## 5. Conclusions

**Figure 8:**
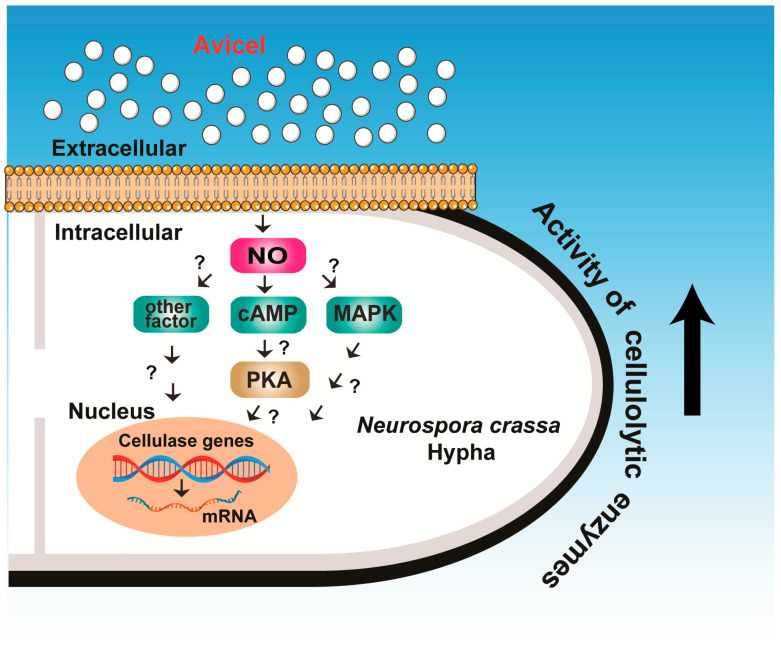
Proposed model for the mechanism of NO in fungal cellulase formation.

Our analysis of the molecular basis of cellulase production in *N. crassa* showed that the intracellular NO levels increased in response to extracellular cellulose, and increased NO levels might stimulate cAMP generation in cells, thereby promoting cellulolytic enzyme transcription (Figure 8).

Our study, to the best of our knowledge, is the first to report the involvement of intracellular NO in fungal cellulase formation. Although the relationship between intracellular NO and downstream signaling pathways was not completely clarified in our study, our preliminary results suggest the potential involvement of the cAMP/PKA, calcium, and MAPK signaling pathways in the regulatory network of cellulolytic enzyme production in *N. crassa* and warrant further intensive investigation. Molecular regulation of fungal cellulase production has been most extensively studied in *T. reesei*. However, these molecular mechanisms are still not completely understood, and extensive studies including various fungal species should be conducted for elucidating the general regulatory network for cellulase production.

## Figures and Tables

**Table 1:** List of primers used in qRT-PCR.

Genes	Primer Sequences
*β-actin*	Forward- 5′-TGA TCT TAC CGA CTA CCT-3′
	Reverse- 5′-CAG AGC TTC TCC TTG ATG-3′
*cbh-1*	Forward- 5′-ATC TGG GAA GCG AAC AAA G-3′
	Reverse- 5′-TAG CGG TCG TCG GAA TAG-3′
*gh6-2*	Forward- 5′-CCC ATC ACC ACT ACT ACC-3′
	Reverse- 5′-CCA GCC CTG AAC ACC AAG-3′
*gh5-1*	Forward- 5′-GAG TTC ACA TTC CCT GAC A-3′
	Reverse- 5′-CGA AGC CAA CAC GGAAGA-3′
*gh3-4*	Forward- 5′-AAC AAG GTC AAC GGT ACG TGG-3′
	Reverse- 5′-TCG TCA TAT CCA TAC CAC TGT TTG-3′

## Data Availability

Not applicable.

## Supplementary Materials

The supporting information can be downloaded at: https://www.mdpi.com/article/10.3390/ijms24054503/s1.
