# Supplementary material for: Intracellular Nitric Oxide and cAMP Are Involved in Cellulolytic Enzyme Production in Neurospora crassa
Source: Int J Mol Sci. 2023 Feb 24;24(5):4503. doi: 10.3390/ijms24054503 (PMC10003064; doi:10.3390/ijms24054503)
Supplement: Supplementary file 1 [file ijms-24-04503-s001.zip › ijms-2210293-supplementary.pdf]

Supplementary Information

Intracellular Nitric Oxide and cAMP Are Involved in Cellulolytic Enzyme Production in *Neurospora crassa*

Nan-Nan Yu [1], Wirinthip Ketya [1], and Gyungsoon Park [1,2,*]

[1] Plasma Bioscience Research Center, Department of Plasma-Bio Display, Kwangwoon University, Seoul 01897, Republic of Korea

[2] Department of Electrical and Biological Physics, Kwangwoon University, Seoul 01897, Republic of Korea

* Correspondence: gyungp@kw.ac.kr; Tel.: +82-2-940-8324

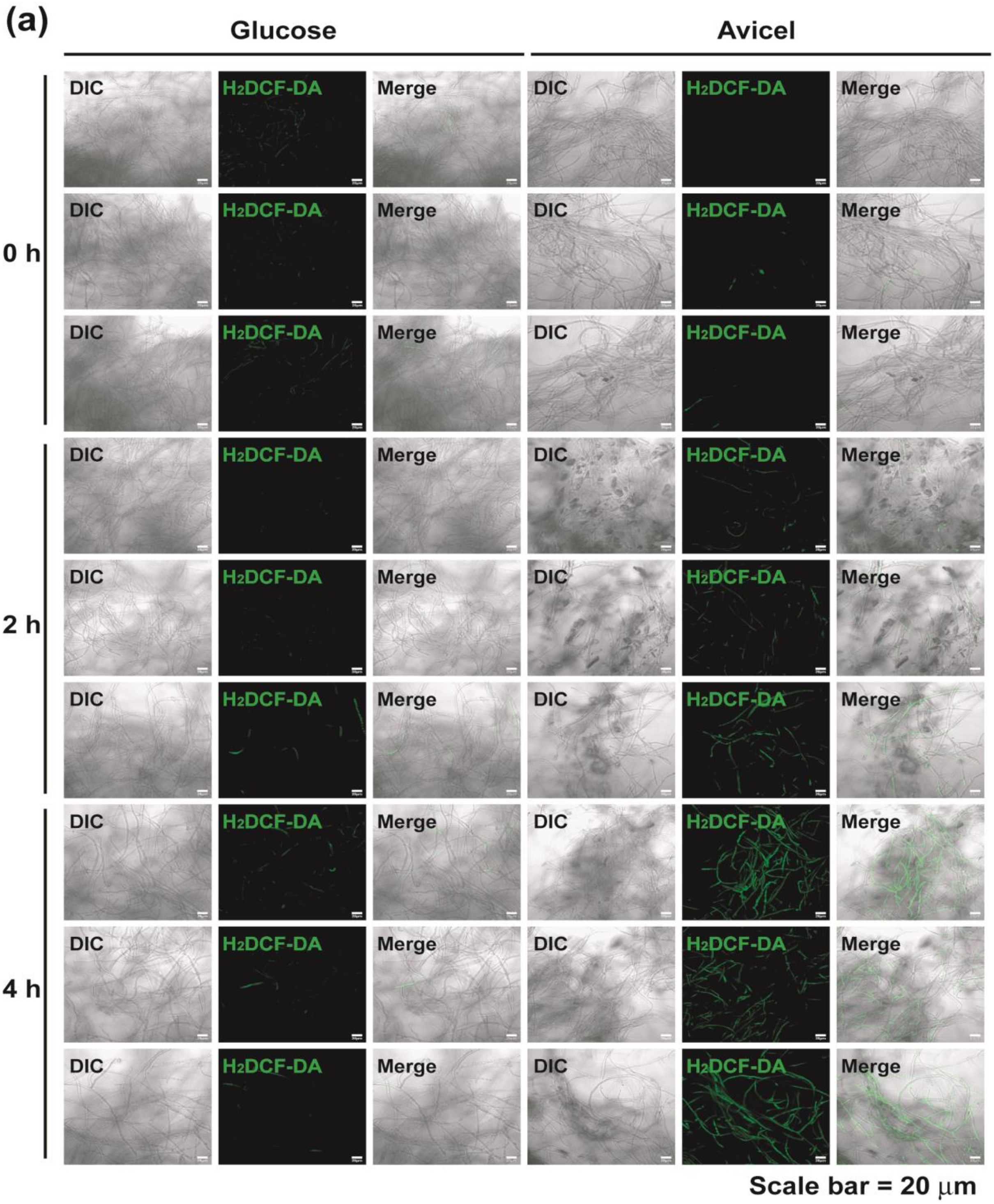
(a)
Glucose
Avicel
0 h
2 h
4 h
DIC
H2DCF-DA
Merge
Scale bar = 20 μm

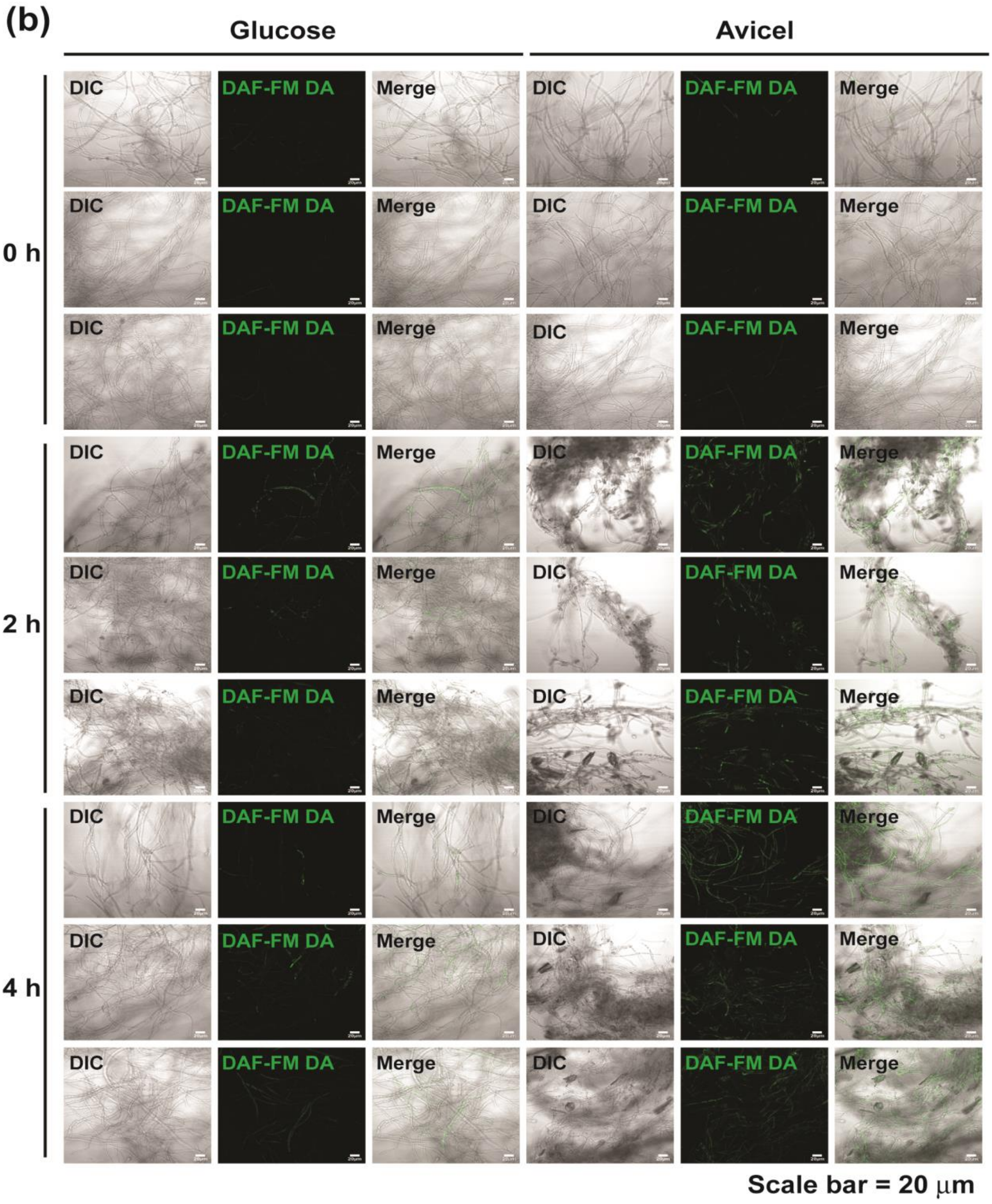

Supplementary Figure S1. Intracellular ROS and NO detected in *N. crassa* hyphae after 0, 2, and 4 h. Fungal hyphae grown in glucose or avicel media for 0, 2, and 4 h were stained with H_2DCF-DA (ROS) **(a)** and DAF-FM DA (NO) **(b)**. Pictures taken in 3 different areas of fungal hyphae are shown. DIC; Differential Interference Contrast, H_2DCF-DA or DAF-FM DA; fluorescence, Merge; combined image of DIC and fluorescence.

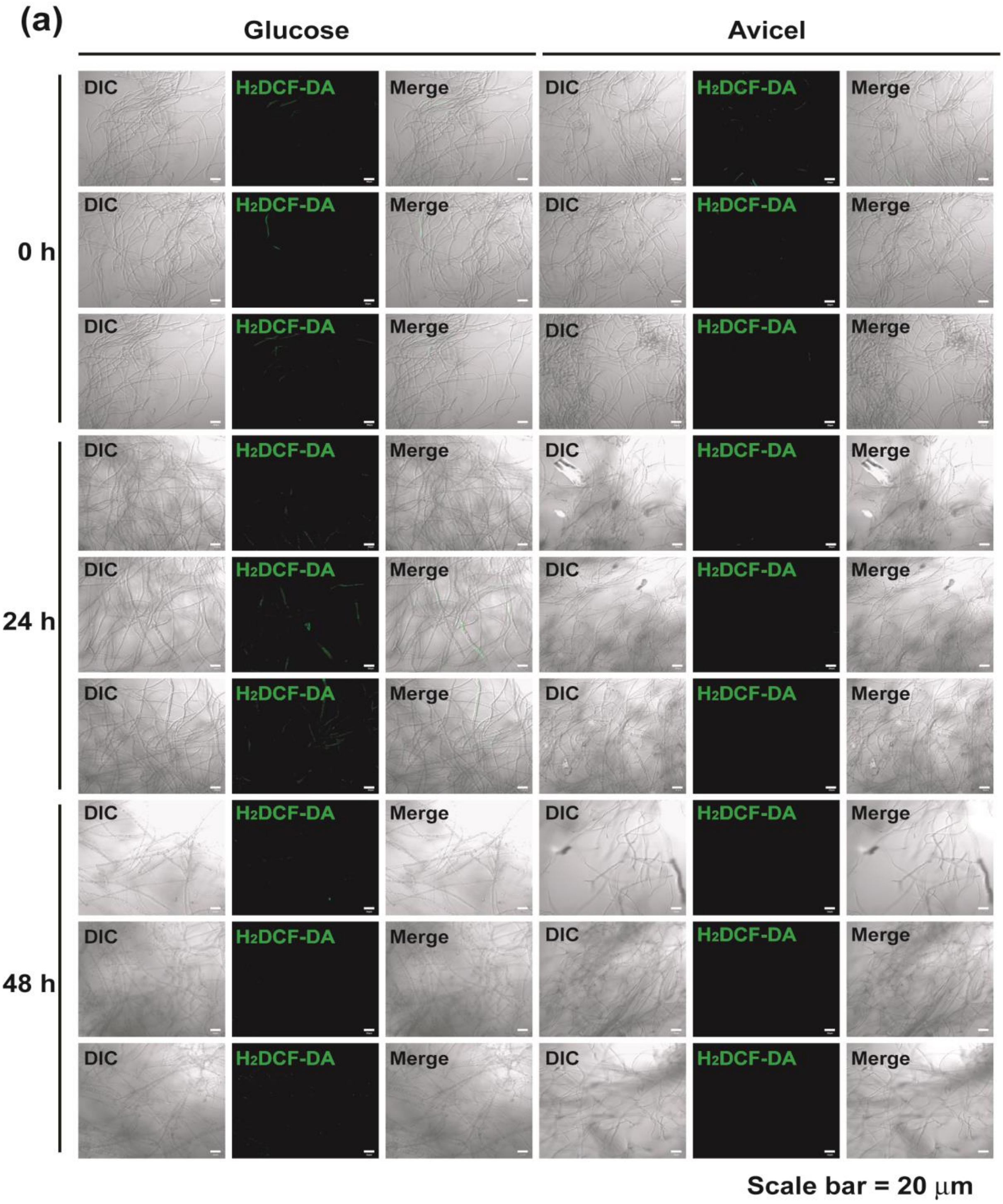

(a)
Glucose
Avicel
0 h
24 h
48 h
DIC
H2DCF-DA
Merge
Scale bar = 20 μm

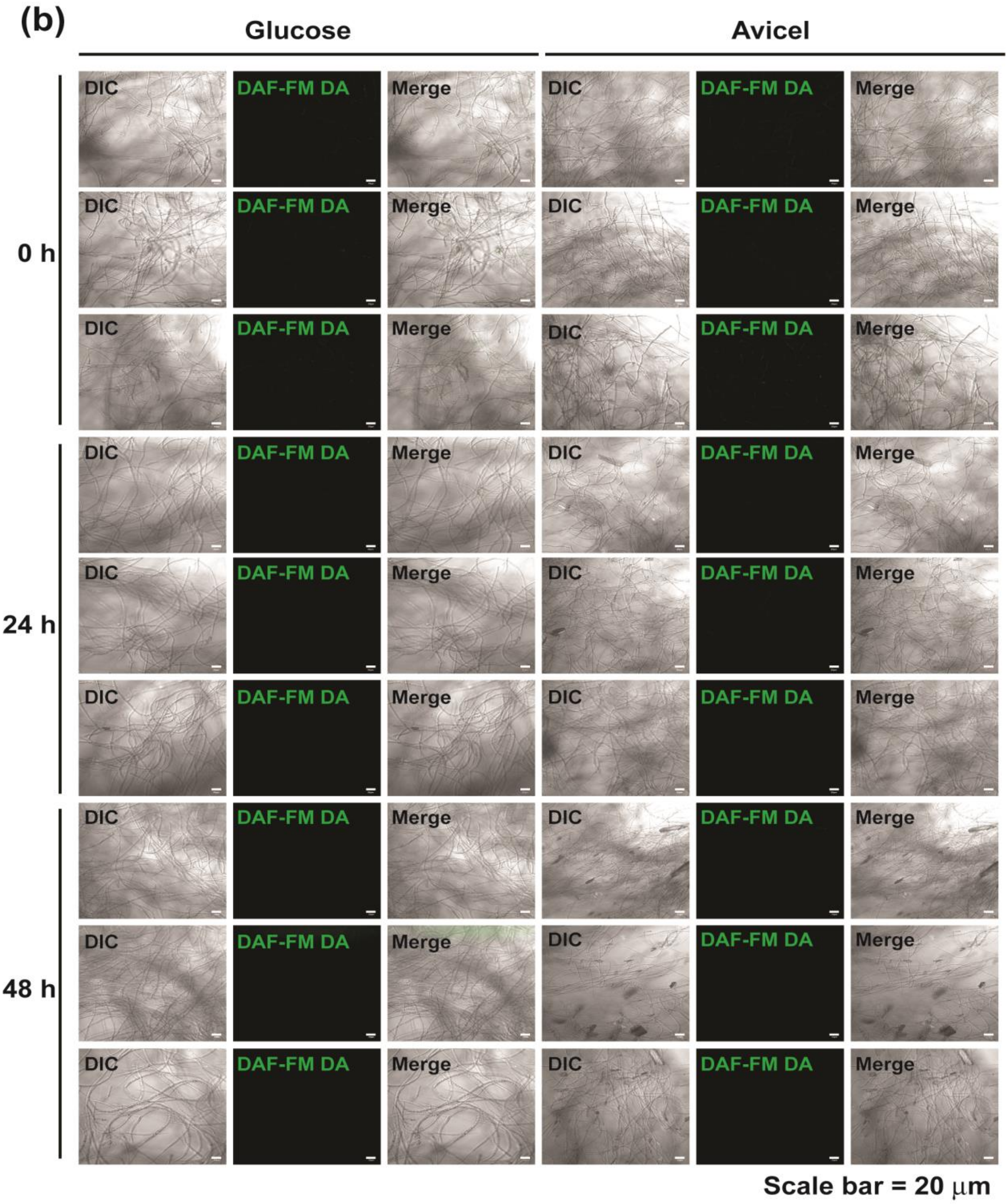

Supplementary Figure S2. Intracellular ROS and NO detected in *N. crassa* hyphae after 0, 24, and 48 h. Fungal hyphae grown in glucose or avicel media for 0, 24, and 48 h were stained with H_2DCF-DA (ROS) **(a)** and DAF-FM DA (NO) **(b)**. Pictures taken in 3 different areas of fungal hyphae are shown. DIC; Differential Interference Contrast, H_2DCF-DA or DAF-FM DA; fluorescence, Merge; combined image of DIC and fluorescence.

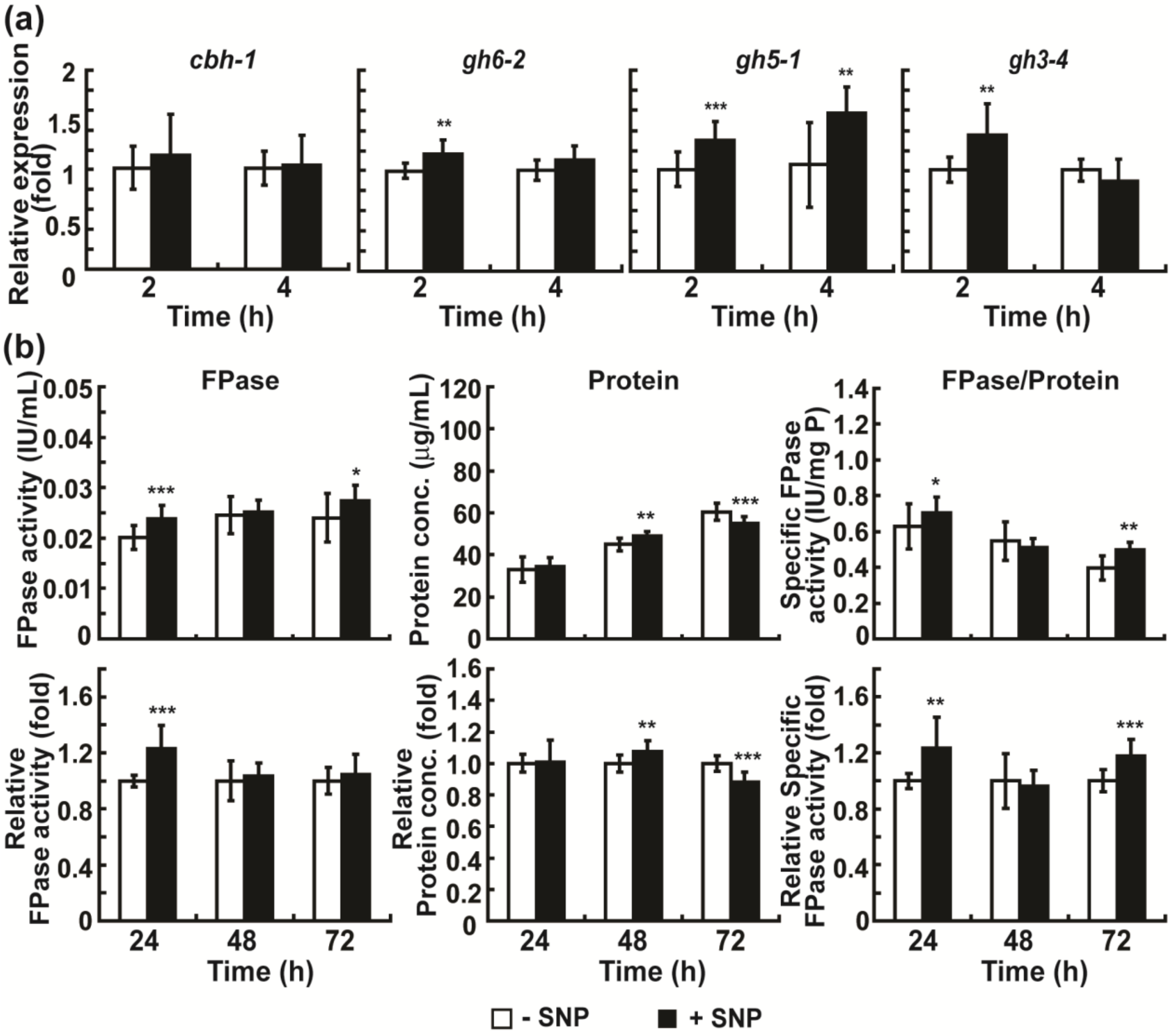

Supplementary Figure S3. **Effect of SNP treatment on production of cellulolytic enzymes in *N. crassa* grown in glucose media.** **(a)** Level of mRNA of cellulolytic enzymes (*cbh1*, *gh6-2*, *gh5-1*, *gh3-4*) in fungal hyphae grown in glucose media with or without SNP for 2 and 4 h. **(b)** Filter paper enzyme activity (total activity of cellulolytic enzymes), total protein concentration, and specific cellulolytic enzyme activity in glucose media with or without SNP for 24, 48, and 72 h. In (a) and (b), fungal hyphae grown in glucose media for 24 h were transferred to glucose media with or without SNP and incubated for 24, 48, and 72 h. – SNP group: only glucose media, + SNP group: 0.01 mM SNP in glucose media. Each value is the mean of 6 or 9 replicate measurements (3 replicates per experiment and 2 or 3 independent experiments): *$p < 0.05$, ** $p < 0.01$, *** $p < 0.001$.

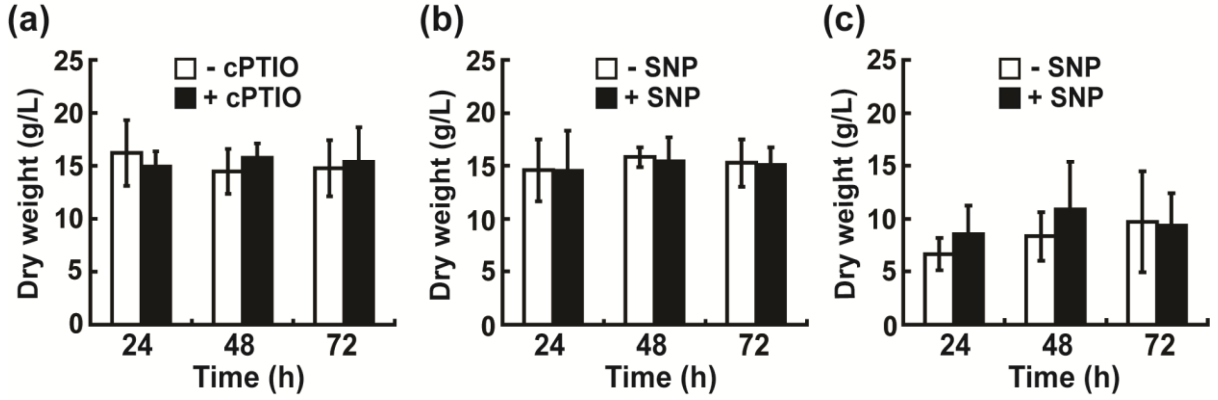

Supplementary Figure S4. **Effect of cPTIO or SNP treatment on fungal biomass.** **(a)** Dry weight of fungal mycelia harvested from culture in avciel media with or without cPTIO for 24, 48, and 72 h. – cPTIO group: only avicel media, + cPTIO group: 10 mM cPTIO in avicel media. **(b)** Dry weight of fungal mycelia harvested from culture in avciel media with or without SNP for 24, 48, and 72 h. – SNP group: only avicel media, + SNP group: 0.01 mM SNP in avicel media. **(c)** Dry weight of fungal mycelia harvested from culture in glucose media with or without SNP for 24, 48, and 72 h. In (a), (b), and (c), fungal hyphae grown in glucose media for 24 h were transferred to avicel (a and b) or glucose (c) media with or without cPTIO and SNP and incubated for 24, 48, and 72 h. Each value is the mean of 6 or 9 replicate measurements (3 replicates per experiment and 2 or 3 independent experiments).

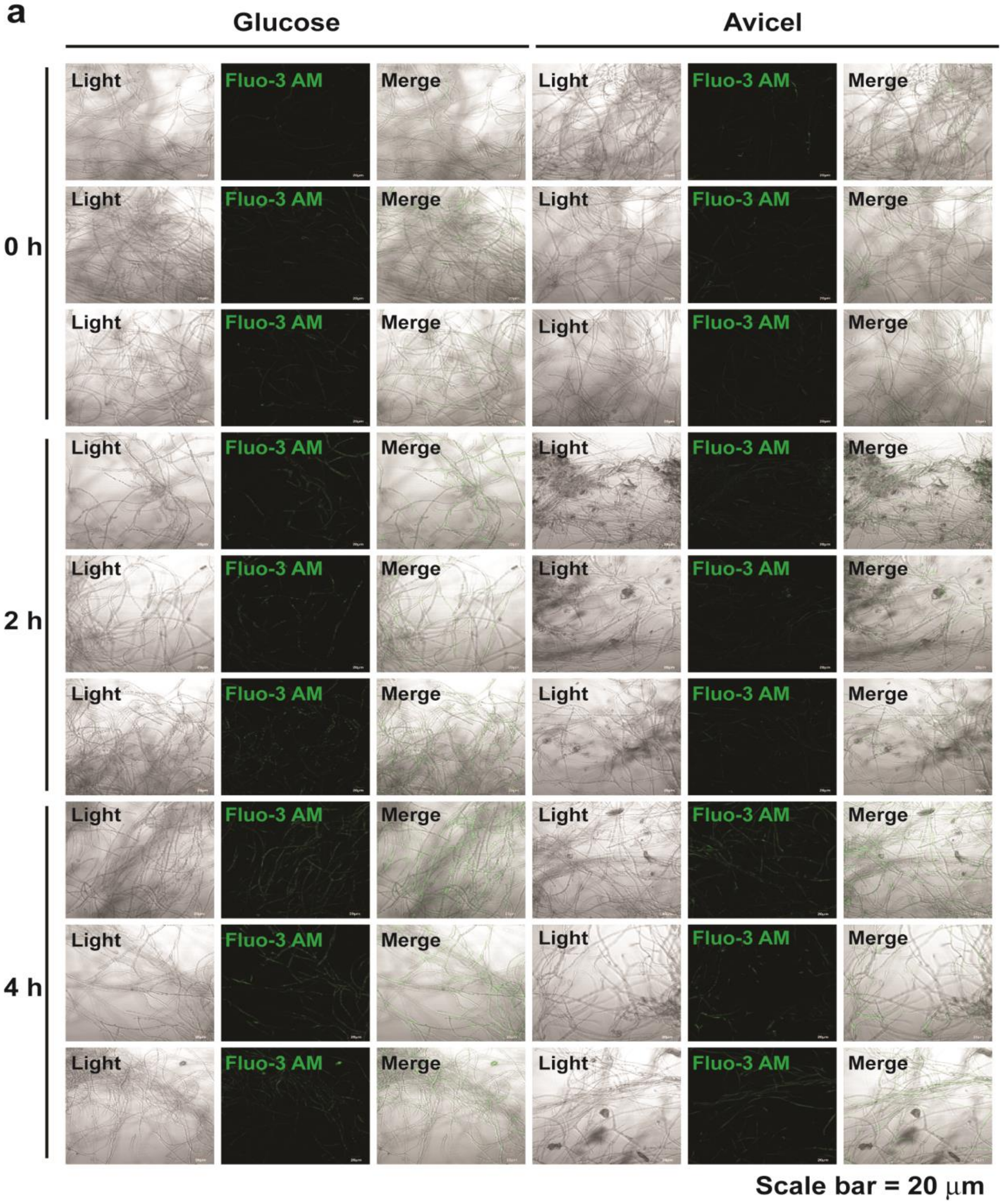

Supplementary Figure S5. Intracellular Ca^{2+} in *N. crassa* hyphae. Fungal hyphae grown in glucose or avicel media for 0, 2 and 4h were stained with Fluo-3 AM (green fluorescence). Pictures taken in 3 different areas of fungal hyphae are shown. DIC; Differential Interference Contrast, Fluo-3 AM; fluorescence, Merge; combined image of DIC and fluorescence.

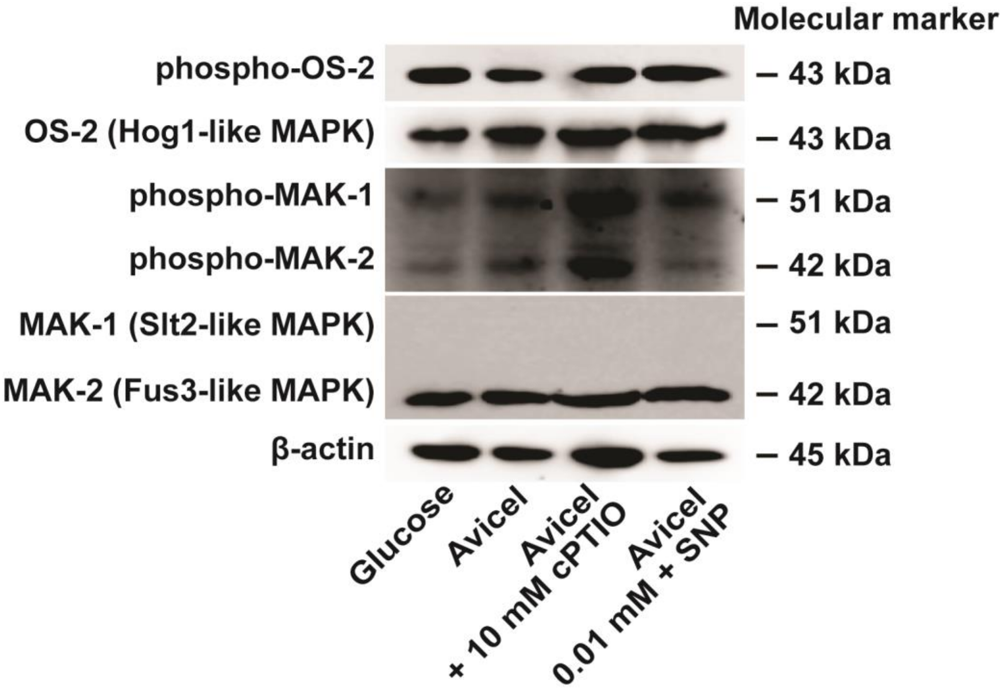

Supplementary Figure S6. Western blot analysis for phosphorylation of MAP kinases after treatment with cPTIO (NO scavenger) and SNP (NO donor). Using total proteins extracted from fungal hyphae grown for 4 h, western blot analysis for protein and phosphorylation of 3 MAP kinases, MAK-1, MAK-2, and OS-2, was performed. β-actin was used as a reference protein. Glucose group: only glucose media; Avicel group: only avicel media; Avicel + cPTIO: avicel media + 10 mM cPTIO; Avicel + SNP: avicel media + 0.01 mM SNP.
